# Phencyclidine Disrupts the Auditory Steady State Response in Rats

**DOI:** 10.1371/journal.pone.0134979

**Published:** 2015-08-10

**Authors:** Emma Leishman, Brian F. O’Donnell, James B. Millward, Jenifer L. Vohs, Olga Rass, Giri P. Krishnan, Amanda R. Bolbecker, Sandra L. Morzorati

**Affiliations:** 1 Department of Psychological and Brain Sciences, Indiana University, Bloomington, IN, United States of America; 2 Department of Psychiatry, Indiana University School of Medicine, Indianapolis, IN, United States of America; 3 Larue D. Carter Memorial Hospital, Indianapolis, Indiana, United States of America; 4 University of California Riverside, Riverside, CA, United States of America; Technion - Israel Institute of Technology, ISRAEL

## Abstract

The Auditory Steady-State Response (ASSR) in the electroencephalogram (EEG) is usually reduced in schizophrenia (SZ), particularly to 40 Hz stimulation. The gamma frequency ASSR deficit has been attributed to *N*-methyl-D-aspartate receptor (NMDAR) hypofunction. We tested whether the NMDAR antagonist, phencyclidine (PCP), produced similar ASSR deficits in rats. EEG was recorded from awake rats via intracranial electrodes overlaying the auditory cortex and at the vertex of the skull. ASSRs to click trains were recorded at 10, 20, 30, 40, 50, and 55 Hz and measured by ASSR Mean Power (MP) and Phase Locking Factor (PLF). In Experiment 1, the effect of different subcutaneous doses of PCP (1.0, 2.5 and 4.0 mg/kg) on the ASSR in 12 rats was assessed. In Experiment 2, ASSRs were compared in PCP treated rats and control rats at baseline, after acute injection (5 mg/kg), following two weeks of subchronic, continuous administration (5 mg/kg/day), and one week after drug cessation. Acute administration of PCP increased PLF and MP at frequencies of stimulation below 50 Hz, and decreased responses at higher frequencies at the auditory cortex site. Acute administration had a less pronounced effect at the vertex site, with a reduction of either PLF or MP observed at frequencies above 20 Hz. Acute effects increased in magnitude with higher doses of PCP. Consistent effects were not observed after subchronic PCP administration. These data indicate that acute administration of PCP, a NMDAR antagonist, produces an increase in ASSR synchrony and power at low frequencies of stimulation and a reduction of high frequency (> 40 Hz) ASSR activity in rats. Subchronic, continuous administration of PCP, on the other hand, has little impact on ASSRs. Thus, while ASSRs are highly sensitive to NMDAR antagonists, their translational utility as a cross-species biomarker for NMDAR hypofunction in SZ and other disorders may be dependent on dose and schedule.

## Introduction

Periodic auditory stimulation, such as a train of clicks or amplitude modulated tones, can elicit the auditory steady state response (ASSR) in the electroencephalogram (EEG) which rapidly entrains to the frequency and phase of the stimulus. ASSRs can test the integrity of auditory pathways and cortex, as well as their capacity to generate synchronous activity at specific frequencies [1–8]. ASSRs to 40 Hz stimulation are reduced in power or phase synchronization in patients with schizophrenia (SZ) compared to healthy controls in most [9–23] but not in all studies [24, 25]. An abnormal 40 Hz ASSR has also been associated with familial risk for SZ, suggesting that the deficit may be associated with vulnerability to the illness [16, 24]. At the circuit level, ASSR deficits have been hypothesized to be associated with *N*-methyl-D-aspartate receptor (NMDAR) abnormalities in SZ [9, 26, 27]. *In vitro* and *in vivo* studies suggest that two major cell types, excitatory principal neurons and fast-spiking, parvalbumin inhibitory interneurons, and two specific receptor types, subtype A of the gamma-amino butyric acid receptor family (GABA_A_) and the glutamatergic NMDAR, are critical for neural synchronization in the gamma frequency range (30 to 80 Hz) [28–31]. Similar circuits may be involved in production of the ASSR at gamma frequencies [1, 9, 13, 27].

NMDAR hypofunction has been hypothesized to contribute to the pathophysiology of SZ [26, 32–35]. NMDAR antagonism by pharmacological agents such as phencyclidine (PCP) and ketamine elicit transient schizophrenic-like positive and negative symptoms in humans and produces schizophrenia-related phenotypes in rodents [31, 33, 35, 36]. Pharmacological or genetic manipulations in rodents could test whether alterations in NMDAR function produce electrophysiological disturbances similar to those observed in patients with SZ. NMDAR antagonists can have a profound impact on spontaneous (basal) gamma oscillations, although these effects may differ by region, frequency and local neural architecture [29, 37–41]. In rodents, acute administration of NMDAR antagonists such as ketamine, PCP and MK-801 usually produce an increase in spontaneous gamma power both for *in vivo* local field potentials and EEG [38, 42–45]. In contrast, e*x vivo* mouse studies [46] and rat studies [40] suggest that chronic exposure to ketamine may suppress spontaneous gamma oscillations. Antipsychotic drugs such as clozapine and haloperidol suppress spontaneous gamma power in mice [42] and rats [47, 48]. Some studies have found that antipsychotic treatment attenuates NMDAR antagonist induced gamma hyperactivity [48, 49], while other studies have not found this effect in rodents [42, 47, 50].

Several recent studies have specifically examined the effect of NMDAR antagonists on the intracranial ASSR in rats. Vohs and colleagues [51] found that ketamine administration to unanesthetized, freely moving rats increased ASSR power and phase synchronization near the auditory cortex, especially at 20, 30 and 40 Hz stimulation, but depressed the ASSR at 50 Hz stimulation. Sullivan and colleagues [52] also found that acute injection of MK-801 (0.1 mg/kg) increased ASSR phase synchronization in the auditory cortex for 20 and 40 Hz stimulation, but not at 10 and 80 Hz stimulation. These results suggest that acute NMDA antagonism might increase ASSRs *in vivo* at frequencies at or below 40 Hz, but not at higher stimulation frequencies in unanesthetized rats. Surprisingly, Sullivan et al found that ASSRs were unchanged after 21 days of daily acute MK-801 injections, suggesting a lack of long term alterations of NMDAR function, or development of tolerance to drug effects. In contrast to the preceding studies in unanesthetized rats, acute MK-801 injection produced a reduction of 40 Hz ASSR power and phase locking at the auditory cortex in urethane-anesthetized rats, which was reversed by administration of nicotine [27]. The differences in the 40 Hz ASSR alteration between these two studies may be related to the presence or absence of anesthesia, since ASSRs in humans are suppressed by anesthesia [53] or sleep [54].

The two experiments in the present study address critical questions regarding the effect of PCP, a potent NMDAR antagonist, on the intracranial ASSR in rats. First, the relationship of dose of a NMDAR antagonist to the ASSR frequency response function has not been characterized. Experiment 1 evaluated how three different acute doses of PCP affect the modulation transfer function of the ASSR in rats. Second, only one previous study [52] has compared acute and continuous subchronic administration of a NMDAR antagonist on ASSRs using MK-801. The present study used a similar design with PCP, and will test whether the same differences between acute and subchronic administration hold for PCP. Third, a wide range of stimulation frequencies is used to map the modulation transfer function of the ASSR in both experiments. Time-frequency analysis was used to differentiate effects of PCP on phase synchrony and to overall ASSR power relative to the pre-trial period [1, 55, 56]. Finally, epidural recordings were obtained from the auditory cortex and at the rat vertex region (crown of the skull) because temporal and dorsal sites appear to index different brain generators [57, 58].

## Materials and Methods

### Ethics Statement

The Association for Assessment and Accreditation of Laboratory Animal Care (AAALAC) accredited the facilities. Protocols were approved by the Institutional Animal Care and Use Committee (IACUC) at Indiana University (reference number 0000003253) in compliance with the guidelines of the National Institutes of Health Guide for the Care and Use of Laboratory Animals. All efforts were made to minimize the number of animals used and pain and suffering. General anesthesia was induced and maintained during the surgical procedures with an isoflurane/air mixture. The methods of euthanasia were consistent with the recommendations of the Panel on Euthanasia of the American Veterinary Medical Association.

### Rodents

Adult male (300 g) Sprague-Dawley rats were purchased from Harlan Laboratories (Indianapolis, IN). Rats were acclimated to the facilities for 7 days prior to being individually housed. Food and water were available ad libitum.

### Electrode Implantation

At 12 weeks old (369 ± 6 g), the rats were anesthetized with isoflurane and positioned in a stereotaxic frame with the skull level. A midline incision exposed the skull’s dorsal surface and a second incision reflected the right temporalis muscle, exposing the temporal bone. Stainless steel screw electrodes were epidurally implanted over the vertex (from bregma: P 4.0 mm, from midline: L 1.0 mm), near the auditory cortex (from bregma: P 4.5 mm, from ridge of temporal bone: V 4.5 mm), cerebellum (ground) and frontal sinus (reference) [59]. Stainless steel lead wires were connected to an Amphenol plug and the entire assembly was secured to the skull with dental cement. Rats were allowed 2 weeks recovery prior to EEG recording.

### Stimulation, Recording and EEG Signal Processing

Rats were unanesthetized and freely moving during the recordings which occurred at the same time of day for each animal across sessions. Rats were allowed 30 min to acclimate to the recording environment. ASSR recordings began 20 min after an injection to allow for drug absorption in treated animals. Thirty 10 s click trains were presented at each of the following frequencies with order randomized across animals: 10, 20, 30, 40, 50 and 55 Hz. Each 10 s click train constituted a trial for subsequent data processing, and there was a 1 s interval between each trial. Clicks (1 ms duration, 90 dB SPL) were delivered via a speaker in front of the animal enclosure. Continuous EEG (bandpass 1–200 Hz) was recorded with a digitization rate of 1000 Hz (Contact Precision Instruments, Cambridge, MA) and saved for off-line processing. EEG was analyzed using Brain Vision Analyzer (Brain Products GmbH, Munich, Germany) and MATLAB (The MathWorks, Natick, MA) code. The 10 s duration of the click train was longer than that typically used in studies of the ASSR in SZ [1].

Raw data were segmented into 10.8 s epochs with a 400 ms pre-trial period and 400 ms post-trial period. Automatic artifact rejection removed trials containing data points outside the range of ±475 μV. Short-time Fourier transform spectrograms were calculated with a moving window of 128 ms, a time step of 10 ms and a pad ratio of 2 for each trial and channel. Phase locking factor (PLF) and mean power (MP) were calculated for each stimulation frequency (± 2 Hz) in the period between 300 ms to 10,000 ms after stimulus onset. PLF and MP measure different attributes of the ASSR (synchronization and power) which may be differentially sensitive to pharmacological manipulations. PLF is the average of normalized phase across trials for every time point and frequency. PLF (also called inter-trial coherence) is a measure of phase synchronization across trials at particular temporal intervals and frequencies. MP (also called event-related spectral perturbation) measures the change in power in a specific frequency band compared to the pre-trial period, thus measuring changes in ASSR power after accounting for basal levels of activity [1, 60]. Unlike PLF, MP measures the amplitude of oscillatory activity independent of phase variability. The MP was computed by first obtaining power for each trial and time point and then subtracting the mean value of the pre-trial period (-400 ms to 0) from entire trial duration.

### Experimental Design

#### Experiment 1: PCP Dose Response Effects

**Fig 1:**
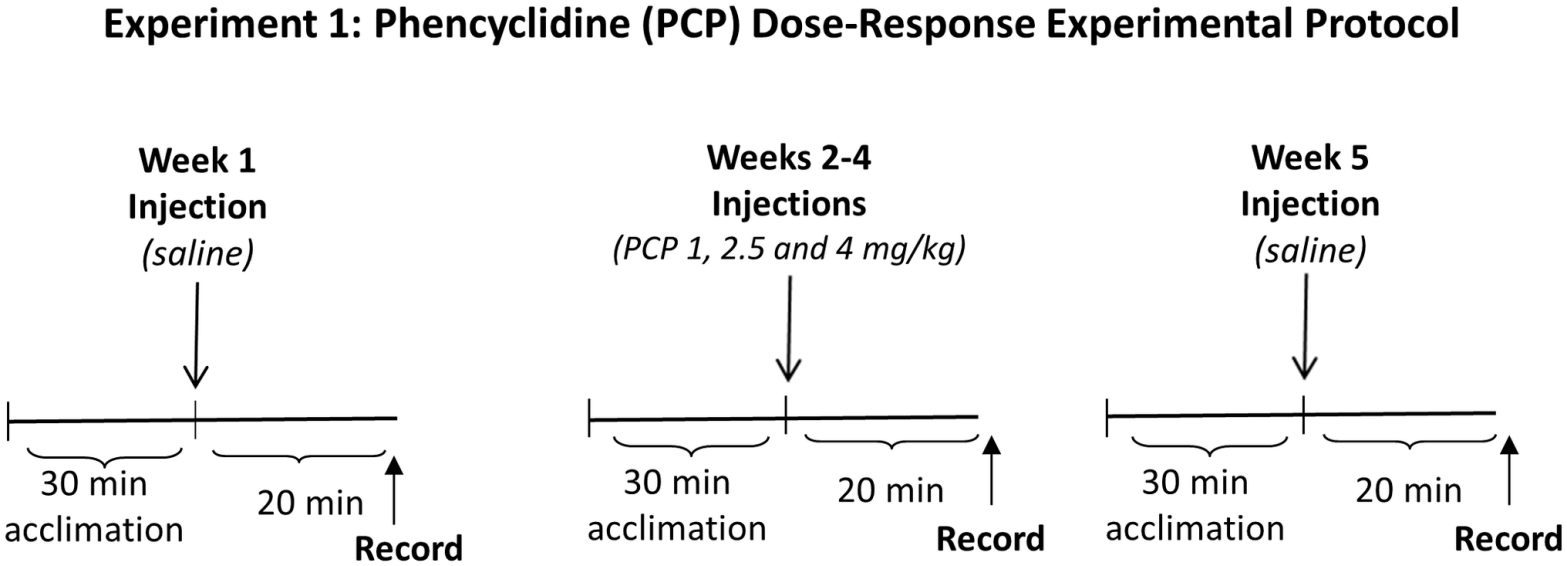
Experiment 1 Design: Effects of acute PCP dose on ASSRs. All rats received saline injection prior to baseline ASSR recordings. After one week, rats received injections of 1.0, 2.5, and 4.0 mg/kg at one week intervals with the order of the three doses randomized among rats. One week after the final PCP injection, washout ASSRs were recorded after a saline injection.

All rats (n = 12) received subcutaneous injections of saline vehicle (pH~7.5) and PCP (pH~7.8). Baseline ASSRs were obtained following a saline injection. One week later, rats began the PCP dosing regimen with injections of 1.0, 2.5, and 4.0 mg/kg one week apart with the order of doses randomly assigned to one of six possible sequences by the investigators prior to the start of the experiment. One week after the final injection, saline was administered and washout ASSRs were obtained. The experimental design for Experiment 1 is shown in Fig 1.

#### Experiment 2: Acute Versus Continuous Subchronic Effects of PCP

**Fig 2:**
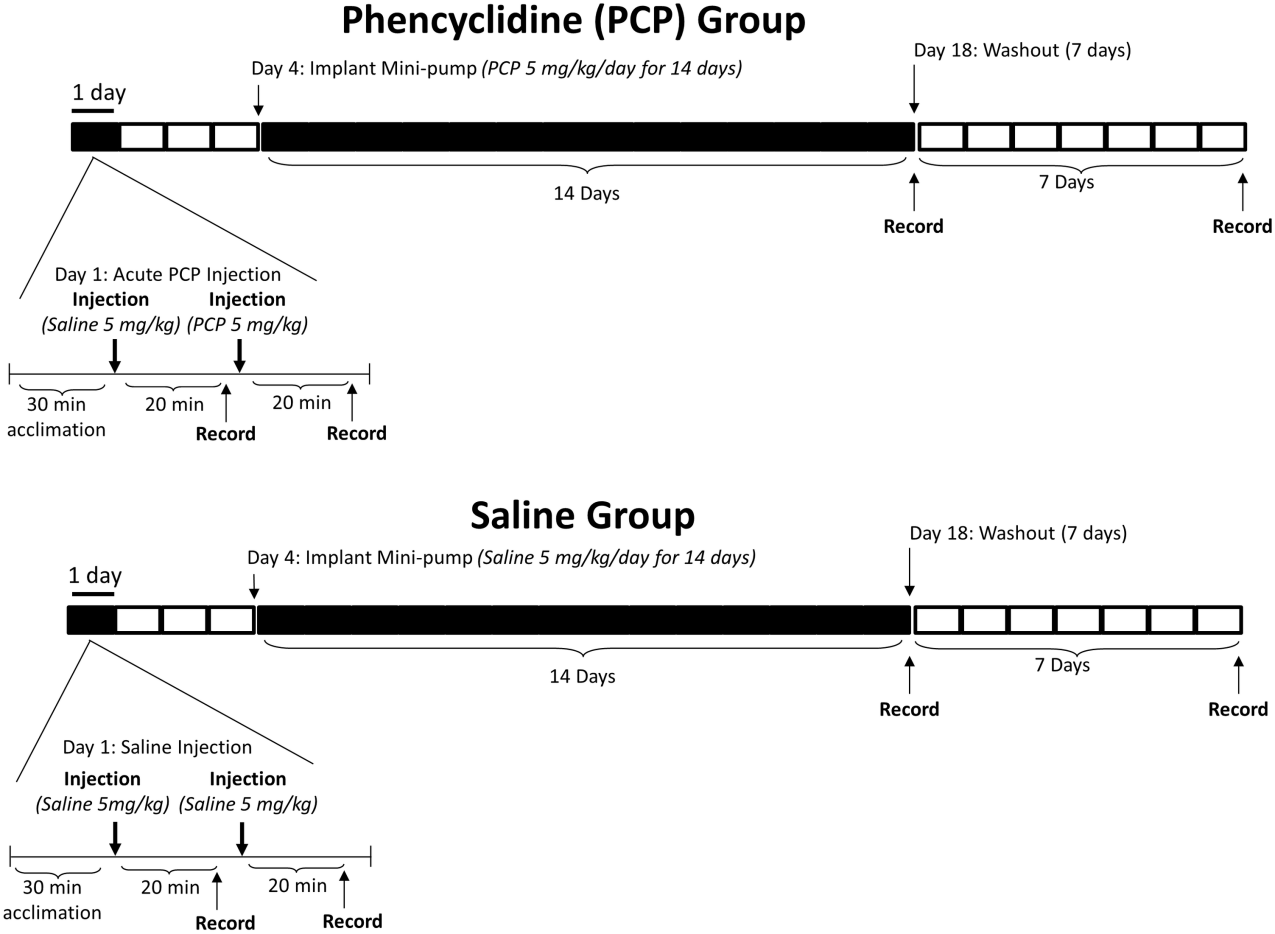
Experiment 2 Design and Timeline: Comparison of ASSRs after acute and continuous subchronic administration of PCP. On day 1, all rats were subcutaneously injected with saline and baseline ASSRs were recorded. Ninety minutes later, control rats were injected with saline while experimental rats received an acute dose of PCP (5 mg/kg). On day 4, each rat was implanted with an osmotic mini pump that subcutaneously delivered either saline or PCP (5 mg/kg/day for 14 days). On day 18, ASSRs were recorded to assess the effect of chronic PCP exposure. After 25 days (7 days after cessation of drug delivery) washout ASSRs were obtained. Black regions in the time line indicate periods when the rats in the PCP arm received PCP.

Rats were randomly assigned to either a PCP (N = 13) or saline control group (N = 11). On day 1, all rats were subcutaneously injected with saline and baseline ASSRs were recorded. Ninety minutes later, control rats were injected with saline while experimental rats received PCP (5 mg/kg). This acute dose produces behavioral disturbances in rats [61]. On day 4, each rat was implanted with an osmotic mini pump (model 2ML2, Duret Corp., Cupertino, CA) between the scapulae that subcutaneously delivered either saline or PCP (5 mg/kg/day for 14 days). Continuous subchronic administration of PCP produces a lower peak serum level than a single acute injection [62–64], with two weeks of continuous PCP administration at 5 mg/kg/day producing serum levels of about 16 ng/ml [63]. Similar subchronic dosages of continuous or repeated PCP administration have produced deficits in extradimensional shift learning [65], novel object recognition [66] and potentiation of amphetamine induced locomotion [63]. On day 18, ASSRs were recorded to assess the effect of subchronic PCP exposure. After 25 days (7 days after cessation of drug delivery) washout ASSRs were obtained. Since PCP half-life in Sprague-Dawley rats has been estimated to be between 30 and 83 minutes in pharmacokinetic studies [67, 68], PCP levels in the brain should therefore approach zero after a one week washout period. The experimental Design for Experiment 2 is shown in Fig 2.

### Statistics

In Experiment 1, repeated measures analysis of variance (ANOVA) with the within subject factors of *Dose* (5 levels: baseline; 1.0, 2.5, and 4.0 mg/kg PCP; washout), electrode (2: auditory cortex, vertex) and *Frequency* of stimulation (6: 10, 20, 30, 40, 50, 55) was used to detect drug effects on PLF and MP. To evaluate interactions which occurred between *Dose* and other factors, follow up ANOVAs were calculated for each stimulation frequency and site (auditory cortex, vertex) with Sidak tests to compare values between baseline and different doses.

In Experiment 2, mixed design ANOVAs with the repeated measure factors of *Time* (4: baseline, acute, subchronic, wash out), *Frequency* of stimulation (6: 10, 20, 30, 40, 50, 55) and *Electrode* (2: auditory cortex, vertex) and the between subjects factor of *Group* (2: PCP, saline) were used to assess PLF and MP. Each frequency of stimulation and site was tested by a separate ANOVA. When interactions involving *Group* and *Time* occurred, follow up repeated measures ANOVAs for specific frequencies of stimulation with the within subjects factor of *Time* (4) were used in conjunction with Sidak tests to identify differences from baseline for each treatment arm. Three rats lacked data in the 55 Hz stimulation condition due to technical problems, and were excluded from the overall ANOVA, but were included in frequency specific analyses.

**Data Files in Supplementary Information:** Data for Experiment 1 is available in the S1 File, and for Experiment 2 in the S2 File for this paper.

## Results

### Experiment 1: PCP Dose Response Effects

#### Overall ANOVAs

Repeated measures analysis of variance (ANOVA) with the within subject factors of *Dose* (5), electrode *Site* (2) and *Frequency* of stimulation (6) was used to detect drug effects on PLF and MP. The ANOVA on PLF showed significant (p < .05) effects and interactions for *Site*, *Site X Frequency*, *Site X Dose*, *Frequency X Dose*, *and Site X Frequency X Dose*. The ANOVA on MP showed significant (p < .05) effects and interactions for *Site*, *Frequency*, *Dose*, *Site X Frequency*, *Site X Dose*, *Frequency X Dose*, *and Site X Frequency X Dose*. The main effect and interactions for the *Site* factor reflected the consistently larger responses at the auditory cortex site compared to the vertex site, similar to findings by Sullivan et al [52]. In order to explicate the two and three way interactions, follow up ANOVAs were carried out for each electrode site and condition for PLF and MP measures. Sidak corrections were used to test for differences between the baseline measure and subsequent recordings.

#### Auditory cortex site

**Fig 3:**
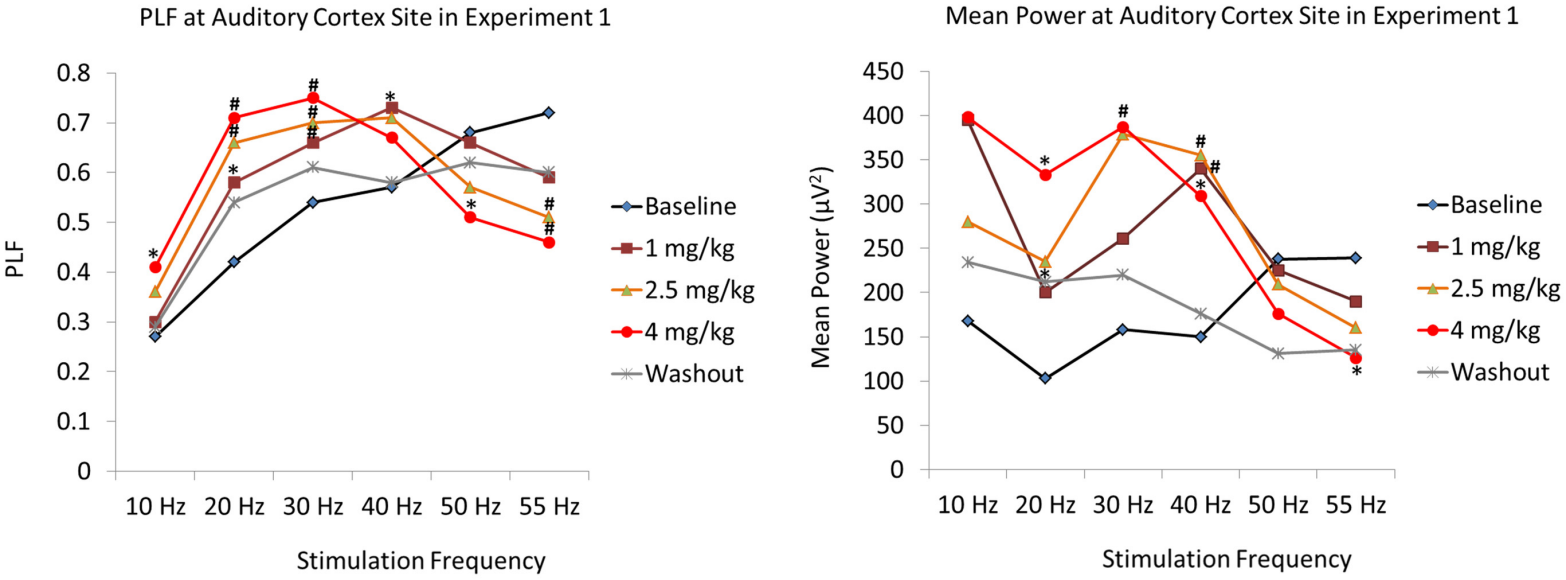
Experiment 1, Dose Response Study. Graphs showing the mean PLF and MP values at each acute dose of PCP as a function of frequency of stimulation at the auditory cortex electrode. Asterisks indicate differences from baseline values (* p≤.05, # p≤.01). PCP caused a dose-dependent increase of PLF at low frequencies of stimulation (10, 20, 30 and 40 Hz), and suppressed PLF at high frequencies (50 and 55 Hz.)

**Fig 4:**
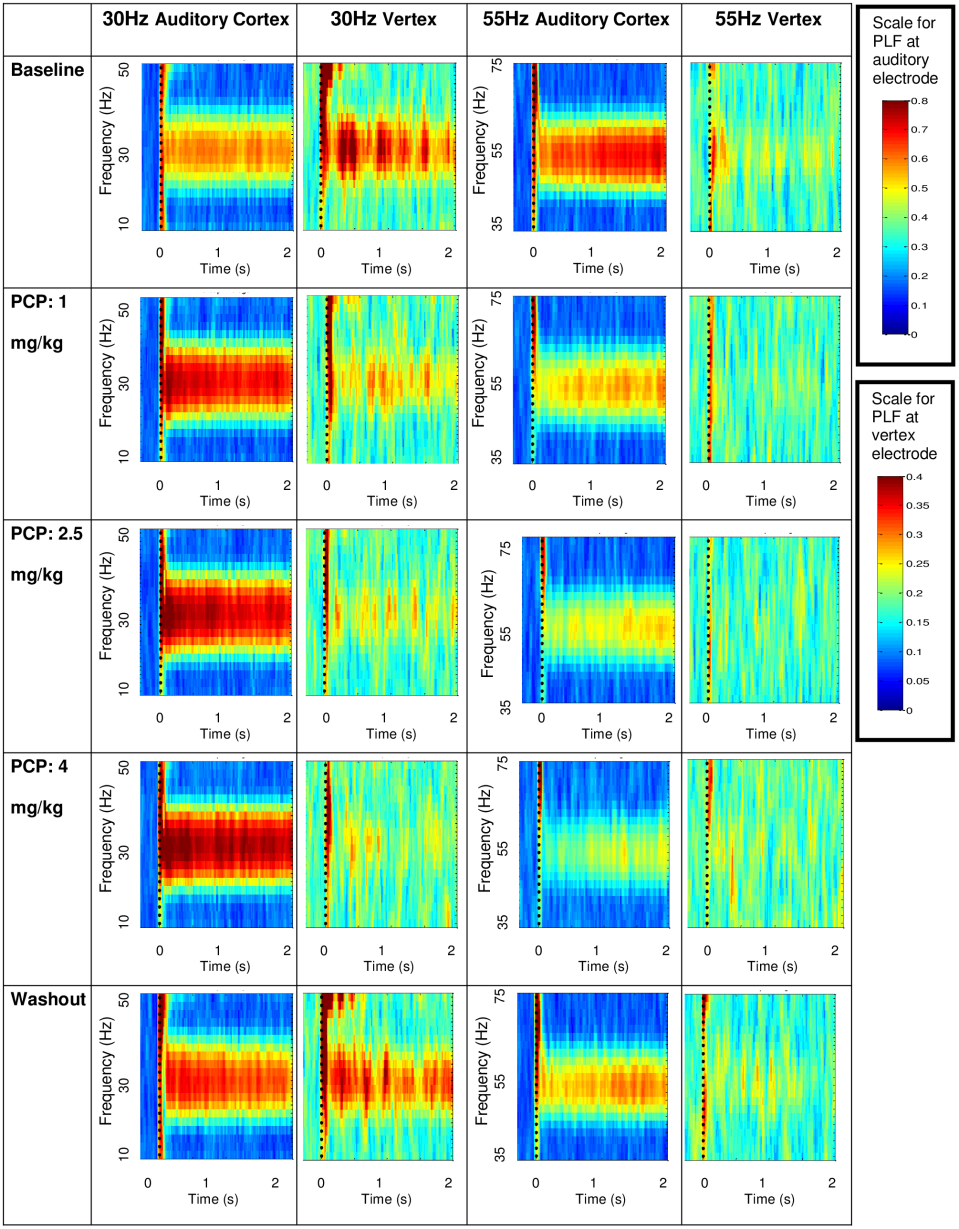
Experiment 1, Dose Response Study. Effect of PCP dose on PLF at 30 Hz and 55 Hz at auditory cortex and vertex sites. Each spectrogram shows the averaged PLF as a function of time and frequency. The spectrogram’s color indicates PLF: the deepest red color represents a PLF value of 0.8 at the auditory cortex electrode and a PLF of 0.4 at the vertex electrode (see scale at right of figure). The vertical dotted line indicates the time of stimulus onset; there was little phase locked activity before the onset of the click train. At the auditory cortex site, PCP increased PLF at 30 Hz, but decreased PLF at 55 Hz stimulation. At the vertex site, PCP decreased PLF for both frequencies of stimulation.

**Phase Locking Factor:** PCP increased PLF at frequencies below 50 Hz, and attenuated PLF at 50 and 55 Hz. An effect of *Dose* indicated that PCP increased PLF for 10 (F(4,44) = 9.26; p < .001), 20 (F(4,44) = 14.75; p < .001, 30 (F(4,44) = 9.98; p < .001) and 40 Hz (F(4,44) = 7.08; p < .001) stimulation. In contrast, PCP caused a decrease in PLF at higher stimulation frequencies, as indicated by a main effect of *Dose* at 50 (F(4,44) = 5.30; p = .001) and 55 Hz (F(4,44) = 14.22; p < .001). 30 Hz PLF remained elevated, and 55 Hz suppressed, at washout. The effects of PCP on PLF for varying stimulation frequencies and dose (1.0, 2.5, 4.0 mg/kg) are shown in Table 1 and Fig 3. The interaction of *Dose* and *Frequency* of Stimulation was most evident at the highest drug dose. These frequency specific effects are apparent in the spectral plots for PLF for 30 and 55 Hz rates of stimulation in Fig 4.

**Table 1:** Experiment 1: Phase locking factor (PLF) and mean power (MP) as a function of PCP dose at the auditory cortex electrode site.

Frequency	Measure	Baseline *M* (*SD*)	1mg/kg PCP	2.5mg/kg PCP	4mg/kg PCP	Washout
10 Hz	PLF†	0.27 (0.09)	0.30 (0.08)	0.36 (0.09)	0.41 (0.12)*	0.29 (0.08)
	MP†	168.17 (139.37)	395.30 (188.75)	279.79 (126.88)	397.61 (249.28)	233.98 (114.64)
20 Hz	PLF†	0.42 (0.14)	0.58 (0.16)*	0.66 (0.13)**	0.71 (0.14)***	0.54 (0.17)
	MP†	102.63 (115.07)	200.02 (176.61)	234.53 (210.93)	332.94 (305.37)*	212.15(135.92)*
30 Hz	PLF†	0.54 (0.16)	0.66 (0.14)**	0.70 (0.18)***	0.75 (0.13)***	0.61 (0.16)*
	MP†	158.19 (100.45)	260.84 (122.15)	378.86 (280.72)	387.47 (180.39)***	219.60 (139.16)
40 Hz	PLF†	0.57 (0.19)	0.73 (0.15)*	0.71 (0.13)	0.67 (0.10)	0.58 (0.21)
	MP†	149.90 (148.16)	340.45 (141.99)**	354.86 (142.56)**	309.21 (189.85)*	176.06 (157.72)
50 Hz	PLF†	0.68 (0.13)	0.66 (0.14)	0.57 (0.17)	0.51 (0.13)*	0.62 (0.16)
	MP†	237.96 (125.40)	225.46 (124.38)	208.65 (162.72)	175.94 (79.51)	131.10 (76.98)
55 Hz	PLF†	0.72 (0.09)	0.59 (0.15)	0.51 (0.14)**	0.46 (0.12)***	0.60 (0.12)*
	MP†	239.48 (119.67)	189.85 (121.76)	160.15 (101.82)	125.72 (77.91)*	135.34 (77.96)

Note: Phase locking factor (PLF) is scaled from 0 to 1, and mean power (MP) is scaled in microvolts^2^. The † in the measure column indicates a significant main effect of PCP dose (p < .05). The asterisk (*) with bold mean and SD values indicates a significant difference relative to baseline, with asterisks indicating *p≤.05, **p≤.01, ***p≤.001.

**Mean Power:** PCP increased MP as indicated by a main effect of *Dose* for 10 (F(4,44) = 5.05; p < .01), 20 (F(4,44) = 2.66; p < .05), 30 (F(4,44) = 7.16; p < .001) and 40 Hz stimulation (F(4,44) = 10.44; p < .001) (Table 1 and Fig 3), and decreased MP at 55 Hz. The most consistent effects were found at the highest dose (4 mg/kg). In contrast, PCP decreased MP at 50 (F(4,44) = 3.14; p < .05) and 55 Hz (F(4,44) = 4.00; p < .01) stimulation, with increasing doses associated with decreasing MP values. At washout, MP remained elevated for 20 Hz stimulation.

#### Vertex site

**Phase Locking Factor:** While the ASSR showed smaller power and PLF values across conditions at the vertex compared to the auditory cortex site, several *Dose* effects were detected (Table 2): PCP increased PLF at 20 Hz for higher PCP doses (F(4,44) = 9.02; p < .001), but decreased PLF at 30 Hz for all doses (F(4,44) = 25.54; p = .001). There was a *Dose* effect at 40 Hz (F(4,44) = 5.70; p < .001), but no PLF value differed from baseline on post-hoc tests. Spectral plots for PLF at 30 and 55 Hz are shown in Fig 4.

**Table 2:** Experiment 1: Phase locking factor and mean power as a function of PCP dose at the vertex electrode site.

Frequency	Measure	Baseline *M* (*SD*)	1mg/kg PCP	2.5mg/kg PCP	4mg/kg PCP	Washout
10 Hz	PLF	0.18 (0.01)	0.18 (0.02)	0.18 (0.02)	0.19 (0.03)	0.19 (0.02)
	MP†	237.23 (169.99)	385.39 (159.26)	242.24 (136.11)	309.87 (194.63)	35.37(125.42)
20 Hz	PLF†	0.19 (0.02)	0.19 (0.03)	0.21 (0.02)	0.22 (0.02)**	0.19 (0.03)
	MP	41.69 (33.13)	34.25 (40.38)	24.34 (32.75)	6.95 (29.86)	35.84 (46.94)
30 Hz	PLF†	0.31 (0.04)	0.25 (0.04)**	0.23 (0.05)**	0.22 (0.04)***	0.32 (0.06)
	MP†	51.19 (22.94)	30.55 (20.00)*	20.42 (23.08)***	-2.97 (31.58)***	42.33 (46.33)
40 Hz	PLF†	0.27 (0.05)	0.28 (0.03)	0.25 (0.04)	0.22 (0.02)	0.26 (0.04)
	MP	27.63 (17.84)	18.12 (15.16)	18.97 (23.14)	12.25 (41.10)	34.63 (23.90)
50 Hz	PLF	0.18 (0.03)	0.20 (0.02)	0.18 (0.02)	0.19 (0.02)	0.19 (0.02)
	MP	10.81 (9.30)	-1.25 (27.26)	-17.26 (36.29)	-1.55 (42.63)	8.84 (18.97)
55 Hz	PLF	0.20 (0.03)	0.19 (0.02)	0.18 (0.02)	0.20 (0.05)	0.20 (0.04)
	MP†	11.91 (11.59)	-13.41 (34.73)	-.92 (29.70)	-13.70 (19.55)**	4.51 (16.09)

Note: The † in the measure column indicates a significant main effect of PCP dose (p < .05). The asterisk (*) with bold mean and SD values indicates a significant difference relative to baseline, with asterisks indicating *p≤.05, **p≤.01, ***p≤.001. Phase locking factor (PLF) is scaled from 0 to 1, and mean power (MP) is scaled in microvolts^2^.

**Mean Power:** PCP decreased MP at 30 Hz (F(4,44) = 6.08; p = .001) and 55 Hz stimulation (F(4,44) = 3.09; p < .05). At 10 Hz, there was an effect of *Dose* which suggested that PCP increased MP (F(4,44) = 3.02; p < .05), but no specific value differed from baseline on post-hoc tests.

### Experiment 2: Acute Versus Subchronic Effects of PCP

#### Overall ANOVAs

The ANOVA on PLF with the within subjects factors of *Time* (4), *Frequency* of stimulation (6) and *Electrode* site (2) and the between subjects factor of *Group* (2: PCP, saline) showed effects or interactions (p < .05) for *Frequency*, *Site*, *Time*, *Frequency X Site*, *Time X Site*, *Frequency X Group*, *Frequency X Time*, *Group X Frequency X Site*, *Group X Site X Time*, *Frequency X Site X Time*, *and Group X Frequency X Site X Time*. The ANOVA on MP showed significant (p < .05) effects and interactions for *Frequency*, *Frequency X Group*, *Frequency X Site*, *Frequency X Time*, *Site*, *Site X Time*, *Time*, *Time X Group*, *Group X Frequency X Time*, *Frequency X Site X Time*, *and Group X Frequency X Site X Time*. In order to characterize the three and four way interactions, repeated measures ANOVAs were carried out for each electrode site and condition for the PLF and MP measures.

#### Auditory cortex site

**Fig 5:**
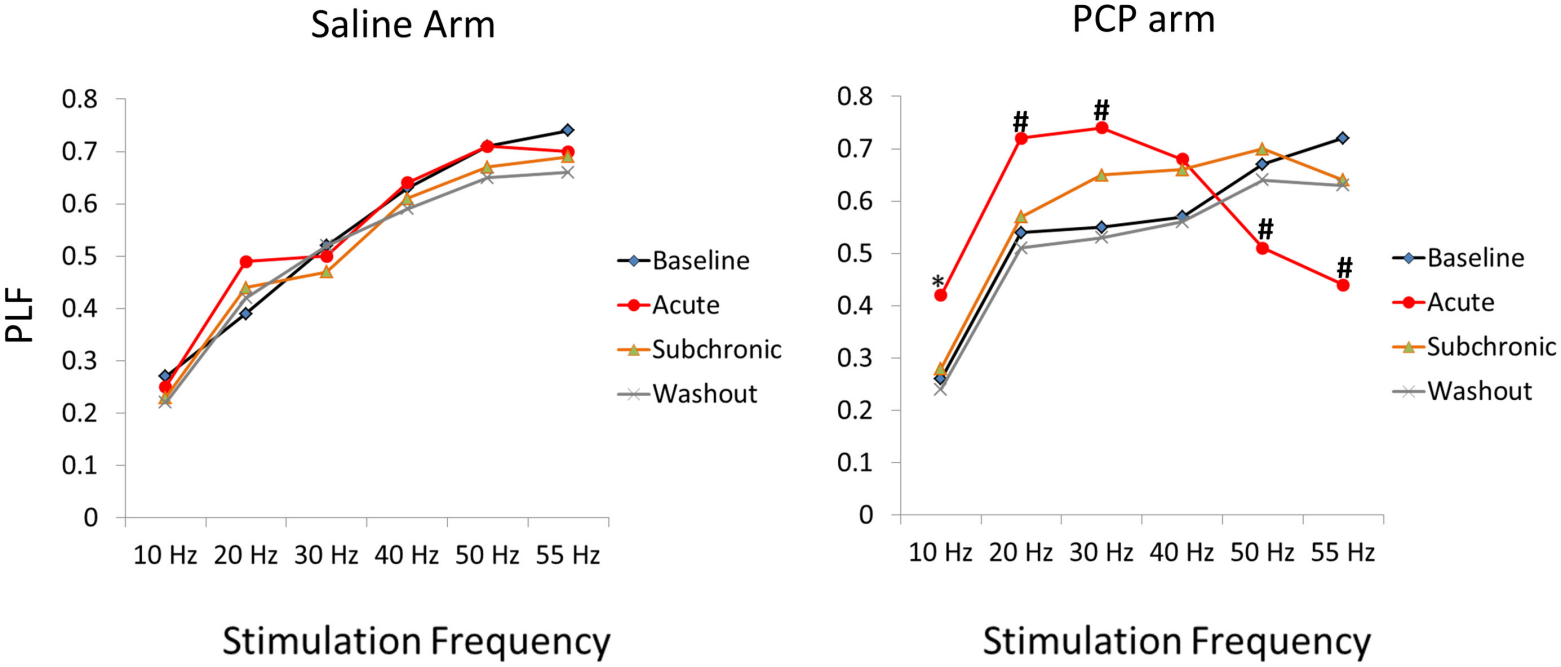
Experiment 2, Acute vs. Subchronic PCP Administration. Graphs showing the mean PLF values for each condition (baseline, acute injections, subchronic administration, washout) as a function of frequency of stimulation at the auditory cortex site. Asterisks indicate differences from baseline values (*p ≤. 05, #p ≤ .01).

**Fig 6:**
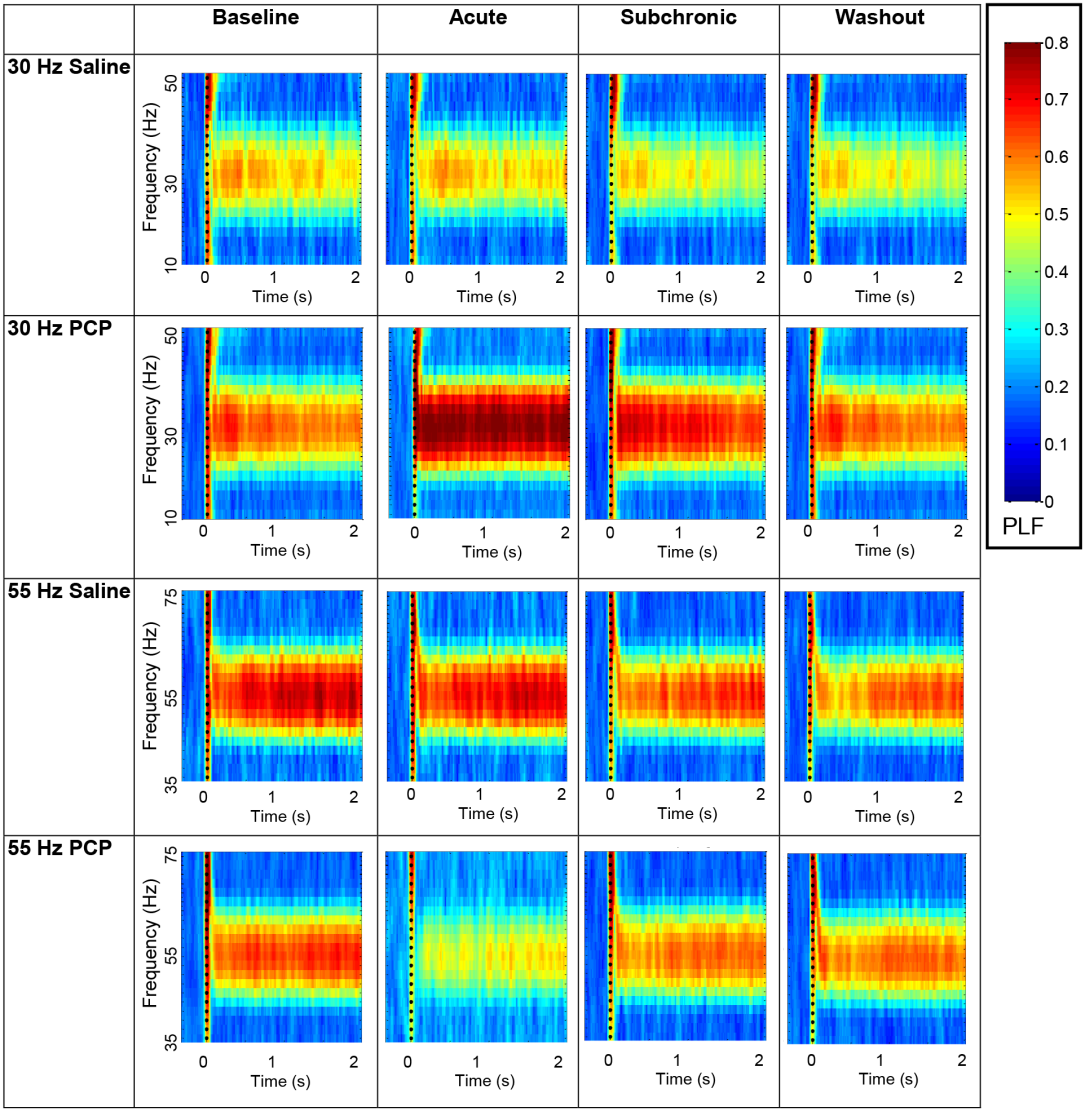
Experiment 2, PLF Time Frequency Spectrograms for Acute and Subchronic Administration. Time frequency spectrograms display the average PLF for the PCP and saline treated rats at baseline, acute injection (5 mg/kg), after two weeks of subchronic administration (5 mg/kg/day), and after a one week washout period. PLF values are shown to 30 and 55 Hz stimulation at the auditory cortex electrode site for 2000 ms after stimulus onset. PCP increased PLF at the acute and subchronic recordings for 30 Hz stimulation, and reduced PLF at the acute recording for 55 Hz stimulation.

**Phase Locking Factor** (Table 3): Acute PCP again decreased PLF at 50 and 55 Hz, while increasing PLF at lower frequencies of stimulation (Figs 5 and 6). The following effects of *Time* on PLF were observed in the PCP treated rats with significant changes from baseline detected by the Sidak test. At 10 Hz there was a significant effect of *Time* (F(3, 36) = 9.80; p < .001). PCP increased PLF from baseline in the acute condition (p = .002), but not in subsequent conditions. At 20 Hz, *Time* was significant (F3, 36) = 17.2; p < .001) indicating that PCP caused an increase in PLF relative to baseline for acute administration (p = .002). At 30 Hz, an effect of *Time* (F(3,36) = 14.02; p < .001) indicated that acute administration increased PLF (p = .001). At 40 Hz, *Time* (F(3,36) = 5.18, p = .004) was only associated with a trend for increased PLF in the acute condition (p = .072). At 50 Hz, *Time* (F(3, 30) = 7.19, p = .001) indicated that PCP reduced PLF in the acute (p = .008), but not subsequent conditions. 55 Hz was associated with an effect of *Time* (F(3,) = 14.16; p < .001) with decreased PLF in the acute condition (p <. 001). In the saline treated rats, there were no effects of *Time* on PLF for any frequency of stimulation.

**Table 3:** Experiment 2: Phase locking factor and mean power at baseline, acute treatment, after subchronic treatment, and after washout for the saline and PCP groups at the auditory cortex electrode site.

Saline Group					
Frequency	Measure	Baseline	Acute	Subchronic	Washout
10 Hz	PLF	0.27 (0.08)	0.25 (0.08)	0.23 (0.07)	0.22 (0.06)
	MP	165.63 (160.82)	168.01 (113.59)	113.68 (133.40)	113.10 (131.57)
20 Hz	PLF	0.39 (0.11)	0.49 (0.10)	0.44 (0.10)	0.42 (0.14)
	MP	71.65 (72.36)	151.77 (77.55)	111.70 (105.73)	115.47 (100.51)
30 Hz	PLF	0.52 (0.21)	0.50 (0.15)	0.47 (0.19)	0.52 (0.19)
	MP	187.52 (205.19)	135.76 (130.21)	126.59 (117.90)	185.09 (150.26)
40 Hz	PLF	0.63 (0.21)	0.64 (0.17)	0.61 (0.21)	0.59 (0.18)
	MP	232.70 (203.97)	240.06 (174.68)	232.98 (192.54)	213.52 (153.19)
50 Hz	PLF	0.71 (0.17)	0.71 (0.12)	0.67 (0.12)	0.65 (0.14)
	MP	271.25 (208.35)	250.17 (165.54)	208.66 (117.57)	193.54 (106.08)
55 Hz	PLF	0.74 (0.15)	0.70 (0.15)	0.69 (0.12)	0.66 (0.09)
	MP†	245.55 (111.78)	205.76 (136.65)	181.15 (92.23)	163.45 (58.70)*
PCP Group					
Frequency	Measure	Baseline	Acute (5 mg/kg PCP)	Subchronic (5 mg/kg/day)	Washout
10 Hz	PLF†	0.26 (0.08)	0.42 (0.19)*	0.28 (0.12)	0.24 (0.05)
	MP†	194.14 (130.82)	479.53 (438.62)	256.85 (132.30)	170.79 (93.09)
20 Hz	PLF†	0.54 (0.15)	0.72 (0.12)**	0.57 (0.16)	0.51 (0.16)
	MP†	168.97 (127.86)	420.71 (286.58)*	232.13 (185.04)	164.69 (125.46)
30 Hz	PLF†	0.55 (0.19)	0.74 (0.15)**	0.65 (0.20)	0.53 (0.18)
	MP†	170.06 (158.29)	471.27 (320.96)**	290.65 (171.19)	162.46 (121.94)
40 Hz	PLF†	0.57 (0.19)	0.68 (0.15)	0.66 (0.22)	0.56 (0.21)
	MP†	172.14 (192.21)	406.70 (249.08)**	305.82 (241.04)	167.52 (151.80)
50 Hz	PLF†	0.67 (0.15)	0.51 (0.14)**	0.70 (0.23)	0.64 (0.23)
	MP	209.78 (131.80)	184.57 (100.09)	297.09 (223.88)	200.32 (183.22)
55 Hz	PLF†	0.72 (0.16)	0.44 (0.12)**	0.64 (0.22)	0.63 (0.20)
	MP	229.09 (154.77)	131.27 (68.57)	192.12 (145.53)	165.75 (132.28)

Note: The † in the measure column indicates an effect of time (p < .05) for a specific measure and frequency. The asterisk (*) with bold mean and SD values indicates a significant difference relative to baseline, with asterisks indicating *p≤.05, **p≤.01, ***p≤.001. Phase locking factor (PLF) is scaled from 0 to 1, and mean power (MP) is scaled in microvolts^2^.

**Fig 7:**
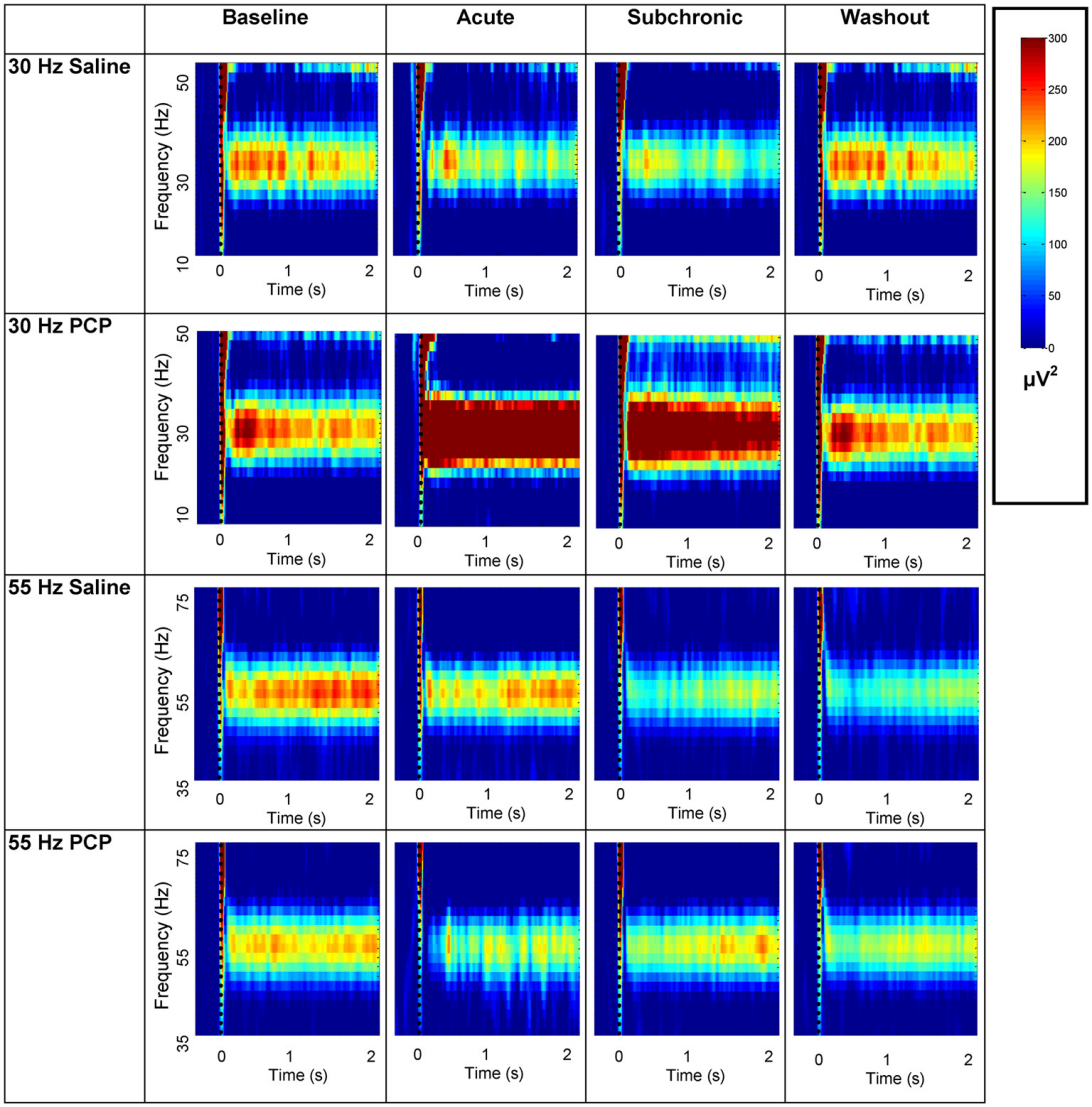
Experiment 2: MP Spectrograms for the Acute and Subchronic Administration. Time frequency spectrograms display the average mean power (MP) for the PCP and saline treated rats at baseline, acute injection (5 mg/kg), after two weeks of subchronic administration (5 mg/kg/day), and after a one week washout period. MP values are shown to 30 and 55 Hz stimulation at the auditory cortex electrode site for 2000 ms after stimulus onset. PCP increased MP at the acute and subchronic recordings for 30 Hz stimulation. Acute PCP decreased MP for 55 Hz stimulation, however, unlike for PLF, the change in MP did not reach statistical significance.

**Mean Power** (Table 3 and Fig 7): Effects of *Time* revealed that acute PCP increased MP from baseline for 10 Hz (F(3,36) = 4.76; p = .007), 20 Hz (F(3,36) = 9.94; p < .001), 30 Hz (F(3,36) = 12.93; p < .001) and 40 Hz stimulation (F(3,36) = 11.73; p < .001). For the saline treated rats, there was a single effect of *Time* (F(3,27) = 3.18; p = .04): MP was reduced at the washout recording (p = .04) but not at the acute or chronic time points.

#### Vertex site

**Phase Locking Factor** (Table 4): Acute PCP administration increased PLF at 20 Hz (3, 36) = 22.83, p < .001), and decreased PLF at 30 Hz (F(3,36 = 8.48; p < .001) and 40 Hz (F(3,36) = 5.13, p = .004). There were no effects of *Time* in the saline treated rats for any frequency of stimulation.

**Table 4:** Experiment 2: Phase locking factor and mean power at baseline, acute treatment, after subchronic treatment, and after washout for the saline and PCP groups at the vertex electrode site.

Saline Group					
Frequency	Measure	Baseline	Acute	Subchronic	Washout
10 Hz	PLF	0.19 (0.02)	0.18 (0.01)	0.18 (0.01)	0.18 (0.02)
	MP	159.01 (170.77)	187.08 (180.06)	157.62 (121.54)	213.48 (94.06)
20 Hz	PLF	0.19 (0.02)	0.20 (0.02)	0.19 (0.02)	0.18 (0.01)
	MP	1.00 (44.12)	-4.57 (75.73)	15.60 (84.46)	5.45 (71.21)
30 Hz	PLF	0.34 (0.04)	0.32 (0.04)	0.33 (0.05)	0.33 (0.06)
	MP	34.23 (35.66)	11.19 (58.25)	32.16 (35.44)	26.42 (53.00)
40 Hz	PLF	0.29 (0.06)	0.29 (0.04)	0.27 (0.06)	0.27 (0.05)
	MP	30.14 (27.91)	24.32 (24.41)	42.26 (35.84)	38.75 (33.48)
50 Hz	PLF	0.19 (0.02)	0.20 (0.04)	0.18 (0.01)	0.19 (0.02)
	MP	11.63 (18.84)	6.93 (9.61)	12.14 (14.71)	8.51 (15.82)
55 Hz	PLF	0.23 (0.04)	0.23 (0.05)	0.22 (0.03)	0.21 (0.03)
	MP	22.68 (23.65)	17.95 (14.70)	10.97 (22.44)	18.33 (15.00)
PCP Group					
Frequency	Measure	Baseline	Acute (5 mg/kg PCP)	Subchronic (5 mg/kg/day)	Washout
10 Hz	PLF	0.18 (0.02)	0.20 (0.05)	0.19 (0.01)	0.18 (0.01)
	MP†	210.81 (108.86)	135.22 (134.12)	259.60 (110.17)	157.49 (93.36)
20 Hz	PLF†	0.19 (0.02)	0.22 (0.03)**	0.18 (0.01)	0.18 (0.01)
	MP	16.02 (36.92)	24.18 (21.92)	29.46 (29.03)	24.27 (40.15)
30 Hz	PLF†	0.30 (0.05)	0.21 (0.02)***	0.31 (0.06)	0.33 (0.13)
	MP†	45.12 (15.36)	5.79 (18.12)**	27.39 (33.38)	55.67 (74.59)
40 Hz	PLF†	0.28 (0.04)	0.22 (0.04)*	0.28 (0.06)	.26 (.04)
	MP	24.69 (29.69)	12.55 (30.36)	23.45 (19.32)	15.70 (27.44)
50 Hz	PLF	0.18 (0.02)	0.18 (0.02)	0.18 (0.01)	0.18 (0.01)
	MP	12.63 (15.38)	-9.08 (24.09)	6.99 (15.79)	2.73 (18.25)
55 Hz	PLF	0.21 (0.04)	0.18 (0.02)	0.19 (0.02)	0.20 (0.03)
	MP	9.83 (17.08)	-0.92 (28.57)	5.74 (12.11)	12.31 (15.71)

Note: The † in the measure column indicates an effect of time (p < .05) for a specific measure and frequency. The asterisk (*) with bold mean and SD values indicates a significant difference relative to baseline, with asterisks indicating *p≤.05, **p≤.01, ***p≤.001. Phase locking factor (PLF) is scaled from 0 to 1, and mean power (MP) is scaled in microvolts^2^.

**Mean Power** (Table 4): At 30 Hz, acute PCP decreased MP compared to baseline (F(3, 36) = 3.52; p = .025). For the PCP arm, there was an effect of *Time* (F(3, 36) = 3.92; p < .05) at 10 Hz, but no value differed from baseline on post-hoc tests. There were no effects of *Time* in the saline treated rats for any frequency of stimulation.

## Discussion

In both experiments, acute PCP injection disrupted the modulation transfer function of the ASSR. Acute administration of PCP increased ASSR activity at lower frequencies of stimulation, and decreased responses at higher frequencies. The suppressive effect of PCP at frequencies that occurred above 40 Hz was most evident for PLF at the auditory cortex site. The magnitude of the effect was dose dependent, with the largest effects at 4 to 5 mg/kg on the PLF measure of phase synchronization. In contrast to an increase ASSR phase synchrony to acute PCP administration, there were no systematic effects of continuous subchronic PCP administration (5 mg/kg/day) on PLF or MP. This replicates the findings of Sullivan’s study, in which there was no effect of 3 weeks of subchronic MK-801 (0.1 mg/kg i.p. daily) treatment on ASSR intertrial coherence at any stimulation frequency [52].

Acute PCP had the most robust effects on the ASSR at the auditory cortex recording site in both experiments. Acute PCP increased MP and PLF at frequencies from 10 to 40 Hz but suppressed MP and PLF at frequencies of 50 and 55 Hz. Sullivan and colleagues reported that acute MK-801 increased intertrial coherence (a measure of phase synchrony equivalent to PLF) for 20 Hz and 40 Hz ASSRs recorded from the auditory cortex of awake rats [69]. A similar loss of high frequency evoked activity was observed to paired click auditory stimuli in transgenic mice with down regulation of the NR1 subunit of the NMDAR [70]. The present study found that baseline corrected power (MP) was often affected by acute PCP administration, while Sullivan et al. [52] did not observe an increase in overall ASSR power to acute MK-801. Given that MK-801 typically increases basal ASSR power, it is conceivable that the dose was not high enough to produce the expected effect.

Experiment 2 again demonstrated that acute PCP injection had robust frequency specific effects, particularly at the auditory cortex recording site. In contrast, subsequent continuous administration of PCP (5 mg/kg/day) had no significant effect on ASSRs. These findings are concordant with accumulating evidence suggesting that acute and subchronic administration of PCP and other NMDAR antagonists can have different effects on measures of behavior, neurochemistry, electrophysiology and neuroanatomy [36, 39]. Acute PCP administration, for example, increases glutamate and dopamine in the prefrontal cortex, while subchronic administration decreases dopamine and glutamate levels [34, 36, 71]. With respect to spontaneous gamma activity, Kittelberger and colleagues [40] found that acute injection of ketamine (10 mg/kg) and MK-801 (0.2 mg/kg) both increased the proportion of gamma oscillations in the hippocampus. In contrast, long term administration of the NMDA antagonist ketamine (30 mg/kg/day for 2 weeks) significantly lowered the proportion of gamma oscillations in the hippocampus freely-moving rats. This change in EEG power was accompanied by a decrease in staining for parvalbumin positive interneurons [40]. Given that effects of acute administration were most evident at the highest doses of PCP, and that continuous administration of PCP produces a lower peak serum level than an acute injection at the same dosage [62–64], it is possible that higher levels of continuous daily administration of PCP would affect ASSRs. In Experiment 1, which used acute injections of PCP at weekly intervals, several ASSR measures remained abnormal after one week washout, also suggesting that repeated acute doses may produce different long term effects on the ASSR than comparable doses delivered through continuous administration.

The cellular mechanisms by which acute PCP produces an increase in low frequency ASSR activity, and dose-dependent suppression of ASSR at frequencies above 40 Hz in rodents, are not yet well understood. The increase in phase synchronization and baseline-corrected power in the present data is concordant with many studies showing increased spontaneous local field potentials and intracranial EEG activity induced by NMDAR antagonists. This increase in low frequency ASSR activity may result from a block on NMDAR receptors on low-threshold spiking interneurons, with resultant glutamatergic hyperactivity (for discussion of this issue, see Kocsis and McCarley [39] and McCarley et al [26]). Consistent with this hypothesis and the broad increase in power induced by NMDAR antagonists, computational modeling by Spencer [72] predicted that reducing NMDAR input to fast spiking interneurons increased network excitability, including gamma power. In mice lacking NMDAR neurotransmission only in fast spiking parvalbumin interneurons, an increase in spontaneous gamma activity, and impaired gamma rhythm induction by optogenetic driving, has also been described [30]. Suppression of gamma range ASSR activity, and a relative increase in 20 Hz activity, is similar to the prediction of the computational model of the effects of a GABA deficit on auditory entrainment by Vierling-Claassen et al [13]. Computational models, therefore, may provide a formal context for interpreting the cellular basis of alterations in the ASSR modulation transfer function in SZ, as well as changes in spontaneous or evoked gamma activity.

While the present data show increased 20 and 30 Hz ASSRs to acute PCP administration and Vohs et al [51] showed similar effects for ketamine, there is little evidence for increases at these ASSR frequencies in SZ spectrum disorders [1]. From a clinical perspective, the relationship of ASSRs to the development and course of illness, treatment, and outcomes in SZ is incompletely characterized, limiting the comparison of rat ASSRs to those observed in SZ patients. It is possible that the premorbid or prodromal period in humans might be associated with increased ASSR activity at lower frequencies of stimulation, similar to the effects of acute PCP administration on ASSRs in rodents. Most SZ patients in previous studies were receiving antipsychotic medication. Since rat studies suggest that antipsychotic treatment can reduce spontaneous EEG [47, 48], a similar effect may occur in humans. However, two studies have reported that the 40 Hz ASSR deficit occurred in non-psychotic relatives of patients with SZ, thus arguing against a primary effect of antipsychotic medication on the 40 Hz deficit [16, 24]. An interesting recent finding by Hirano et al. [23] indicated that while PLF for the 40 Hz ASSR was reduced in SZ, induced (non-phase locked) gamma power was reduced in SZ.

From a methodological standpoint, these experiments indicate that the effects of PCP on ASSRs are highly sensitive to multiple factors, including the specific driving frequency, the dose of the drug, the location of the intracranial electrode, and whether a given dose of the drug is administered acutely or on a continuous schedule. The differential effect of acute PCP on ASSRs elicited by low and high frequencies of stimulation, for example, was most evident at the highest PCP dose, and was more robust for PLF than MP. Our data confirms the observation by Sullivan et al [52] that ASSRs from the auditory cortex recording site appear more sensitive to NMDAR antagonist effects. While we did not compare different types of NMDAR antagonists, it is possible that the effect of acute PCP on ASSR power in our study was specific to PCP, since Sullivan et al. did not observe an effect of MK-801 on ASSR power.

In conclusion, ASSRs appear to be highly sensitive to acute administration of a NMDAR antagonist as suggested by the present data and previous studies [27, 51, 52]. Parallel use of ASSR paradigms in humans and rodent models thus provide a powerful translational vehicle for testing putative cellular mechanisms and development of novel treatments targeting these circuits [31, 39, 73, 74], for understanding of the effects of abuse of NMDAR antagonists and clinical syndromes affecting NMDAR function such as anti-NMDA encephalitis [75]. Important limitations of the current study include the lack of histopathological data, quantification of movement activity or more complex behaviors, lack of cellular recordings, and the use of single, rather than multiple dose levels in the subchronic experiment. Cellular recording and histopathological assays in future studies coupled with computational modeling will help better characterize the cellular and circuit mechanisms that mediate pharmacological effects on the ASSR.

## Supporting Information

S1 File S1_Experiment1_dose_response data. This file contains mean power and PLF values for all rats in Experiment 1. The variables are coded in a Microsoft Excel file in which the columns are variables and the rows are individual rats. The variables (fields) are coded using the following key: Sub_ID = rat identification number; Baseline = initial recording, PCP1 = 1.0 mg/kg, PCP25 = 2.5 mg/kg, PCP4 = 4.0 mg kg, Washout = washout recording; T = auditory cortex (temporal) site, V = vertex site; xHZ = frequency of click train stimulation; MTP = mean power (microvolts^2^), PLF = phase locking factor.
(XLS)

S2 File S2_Experiment2_acute chronic washout data. This file contains mean power and PLF values for all rats in Experiment 2. The variables are coded in a Microsoft Excel file in which the columns are variables and the rows are individual rats. The variables (fields) are coded using the following key: ID = rat identification number;Group (1 = PCP; 2 = saline); Acute = recording after injection of saline or PCP (5 mg/kg), Chronic = recording after two weeks of subchronic, continuous administration (5 mg/kg/day) of PCP or saline; Washout = recording after one week without PCP or saline administration; xHZ = frequency of click train stimulation; T = auditory cortex (temporal) site, V = vertex site; MTP = mean power (microvolts^2^), PLF = phase locking factor.
(XLS)
